# Evaluating the cognitive impact of exergames on community-dwelling older adults beyond laboratory settings: a systematic review and meta-analysis

**DOI:** 10.3389/frdem.2026.1768487

**Published:** 2026-04-10

**Authors:** Yijun Qian, Anna M. Schwartz, Yichi Zhang, Arthur F. Kramer, Alexandre Dias Lopes, Leanne Chukoskie

**Affiliations:** 1 Department of Physical Therapy, Movement and Rehabilitation Science, Bouvé College of Health Science, Northeastern University, Boston, MA, United States; 2 College of Art, Media and Design, Northeastern University, Boston, MA, United States; 3 Beckman Institute, University of Illinois, Urbana, IL, United States; 4 School of Health Professions, Hunter College, The City University of New York, New York, NY, United States

**Keywords:** cognition, exercise, exergame, meta-analysis, older adults, serious game

## Abstract

Cognitive decline in older adults is a growing global concern, with over 55 million people living with dementia worldwide. Exercise games, which combine physical exercise with cognitive training in an interactive digital environment, represent a promising approach for mitigating this decline in community settings. Here we present a systematic review and meta-analysis examining the cognitive effects of exercise games on community-dwelling older adults, including those with mild cognitive impairment and dementia. Across 10 randomized controlled trials with 690 participants (mean age 74 years), exercise games produced a small but statistically significant overall cognitive benefit compared to control conditions (Hedges' g = 0.135, 95% CI [0.037, 0.229], *p* = 0.012). Domain-specific analyses revealed memory showing a trend toward improvement (g = 0.179, *p* = 0.060), while global cognition, executive function, and attention did not reach statistical significance. A single study examining processing speed showed a positive effect, though this finding requires replication. Multicomponent exercise programs incorporating aerobic elements demonstrated numerically larger, though non-significant, effects (g = 0.291, *p* = 0.138) compared to aerobic-only interventions (g = 0.119, *p* = 0.142). The evidence base for clinical populations remains limited and inconclusive, with only two studies examining MCI (g = 0.256, *p* = 0.168) and three studies examining dementia (g = 0.005, *p* = 0.975), neither showing statistically significant effects. These findings provide preliminary evidence supporting exercise games as a modest, accessible supplementary intervention for cognitive health in community settings, though the small effect sizes and limited evidence in clinical populations indicate that substantially more research with larger samples and standardized protocols is needed to establish efficacy and optimize implementation.

**Systematic review registration:**

https://doi.org/10.17605/OSF.IO/AM8QT.

## 1 Introduction

Aging-related cognitive decline has raised significant concerns worldwide, and this decline typically affects areas such as memory, attention, processing speed, and executive function. According to the WHO's 2023 dementia report, over 55 million people are living with dementia, with nearly 10 million new cases emerging each year (World Health Organization, 2023). As the global population ages, this increase contributes to the global burden of disease (Nichols et al., 2022), affects individuals' quality of life, and heightens the stress experienced by informal caregivers within families (Chiao et al., 2015), and substantial economic costs for healthcare systems and societies (Nichols et al., 2022; Wimo et al., 2017).

Current pharmaceutical treatments for cognitive impairment focus primarily on symptom relief without modifying the underlying disease (Srivastava et al., 2021). Researchers have explored various non-pharmaceutical interventions, such as lifestyle interventions, aimed at preventing or slowing cognitive decline and enhancing cognitive performance in older adults. Several lifestyle interventions, such as social engagement, nutrition, and sleep hygiene, have also been implicated in maintaining cognitive health (Kivipelto et al., 2018). These lifestyle interventions have the advantage of fewer side effects and can be used alongside pharmaceutical treatments without adverse interactions (Sikkes et al., 2021).

Aerobic exercise (AE) and cognitive training (CT), in particular, have shown robust benefits for cognitive function across the lifespan (Erickson et al., 2019; Smith et al., 2009). While physical exercise and cognitive training have often been studied separately as single-domain approaches, numerous studies have demonstrated that the combination of exercise and cognitive training is more effective in enhancing cognitive function than either exercise or cognitive training alone (Schaefer and Schumacher, 2010; O'Dwyer et al., 2007; Law et al., 2014; Wollesen and Voelcker-Rehage, 2014). In recent years, there has been a growing trend of using games to combine AE and CT training (Law et al., 2014; Ghai et al., 2017; Mancioppi et al., 2021; Qian et al., 2024). Game-based training programs provide interactive systems (Lumsden et al., 2016) and timely feedback (Kappen et al., 2016), making training more accessible and available to use for older adults in both clinical and home settings (Hill et al., 2017; Gavelin et al., 2021; Zhao et al., 2020).

Exercise games, which are interactive games that require physical movements to play, represent a popular and engaging form of combined physical-cognitive training (Mueller et al., 2018). These games often incorporate elements of balance, coordination, and cognitive challenges within a virtual environment. Exercise games have been increasingly utilized as a physical-cognitive tool due to their interactive nature, which can seamlessly integrate physical and cognitive training together in an entertaining way. Their effectiveness is supported by a series of combined physical and cognitive training studies (Law et al., 2014; Ghai et al., 2017; Mancioppi et al., 2021). Commercial gaming platforms like the *Nintendo Wii* and *Xbox Kinect* have been shown to demand physical effort comparable to traditional moderate exercise (Miyachi et al., 2010; Lyons et al., 2011; O'Donovan et al., 2012; Taylor et al., 2012). Moreover, recent virtual reality exercise games, such as the *Supernatural* boxing-based workout, have demonstrated even higher energy expenditure than traditional game platforms (Gomez et al., 2018; Craig et al., 2024). Beyond the physical benefits of exergaming, which is generally defined as a digital game in which the outcome of the game is predominantly determined by physical effort (Mueller et al., 2011), older adults also experience cognitive gains from the game (Anguera et al., 2022). Various systems have been used for exercise game interventions, and studies have consistently shown that these systems offer equal or superior cognitive benefits compared to single-domain and combined physical-cognitive training (Howes et al., 2017; Stanmore et al., 2017).

Although early evidence on exercise games' cognitive benefits is promising, it is mainly derived from lab or clinical settings. To address the growing demand for community-based interventions and build on the existing evidence base, we conducted a systematic review and meta-analysis to examine the available evidence from community-based studies beyond lab settings. In this meta-analysis, we investigate the overall impact of exercise games on cognition for community-dwelling older adults, which includes both non-clinical and clinical populations (e.g., mild cognitive impairment and dementia). By examining the influence of exercise game characteristics, clinical- and non-clinical population, this study seeks to provide valuable insights for designers, developers, and researchers.

## 2 Method

We adhered to the Cochrane Handbook for Systematic Reviews of Interventions for data processing and analysis in the meta-analysis. Additionally, we followed the Preferred Reporting Items for Systematic Reviews and Meta-Analyses (PRISMA; www.prisma-statement.org) guidelines for reporting our findings. The review is registered on the Open Science Framework (OSF) https://doi.org/10.17605/OSF.IO/AM8QT.

### 2.1 Search strategy

A systematic electronic database search was conducted to identify relevant published studies. The PubMed, ScienceDirect, SpringerLink, ACM, IEEE databases were included in our search to identify relevant studies published from Jan, 2014 to June, 2024.

For this review of the impact of exercise games on cognitive function in older adults, a comprehensive literature search was conducted using specific keywords and Boolean operators to ensure a detailed and focused retrieval of relevant studies. The search terms included combinations of (“exercise game" OR “active video game" OR “virtual reality" OR “augmented reality") to capture various forms of interactive and immersive exercise technologies. These were paired with cognitive function-related terms (“attention" OR “memory" OR “executive function" OR “processing speed") to target studies examining specific cognitive outcomes. Additionally, the search was refined to the population of interest using terms like (“older adults" OR “seniors" OR “elderly"). The detailed search terms for each database are described in Supplementary material Data Sheet 1.

### 2.2 Study selection

Our meta-analysis only included studies published in peer-reviewed journals or at conferences. Posters presented at peer-reviewed conferences with an accessible full conference paper were also included. Protocols and literature reviews were excluded. Study participants included cognitively healthy older adults or older adults with cognitive impairments, such as Mild Cognitive Impairment (MCI) and different types of Dementia, and the mean age of participants had to be at least 60 years old. The studies included at least one exercise game training group, and studies were considered only when they included an active (e.g., yoga, running, biking) or non-active control group (e.g., educational material) in addition to the exercise game interventions group. Studies with interventions that were irrelevant to exercise game interventions were excluded from this meta-analysis. Studies needed to include at least 12 weeks of intervention. This threshold was chosen based on its widespread use in interventional studies focused on cognitive improvement in older adults (Ngandu et al., 2015; Dedeyne et al., 2017). Studies have demonstrated that cognitive changes are typically observable with 12 or more weeks to produce reliable and sustained measurements on cognitive improvements (Dedeyne et al., 2017). Studies with the acute effects of an intervention (e.g., after single training sessions) were excluded. Included studies could be randomized controlled trials (RCT) or cluster-RCT, and training had to take place in home-based or community-based environments (e.g., daycare center, nursing home, and other residential places), while lab-based or hospital- or clinic-based studies were excluded. Finally, all included studies must have the necessary data to compute Hedges' g, such as sample size, mean, standard deviation, and standard errors for at least one cognitive outcome, and the study had to include at least one cognitive measurement, which could be global cognition, attention, memory, executive functions, verbal learning, or processing speed.

### 2.3 Data extraction

Articles searched from electronic databases were organized into an Excel sheet with titles and abstracts for initial screening by two researchers. Two researchers (YQ and AS) extracted data independently from eligible studies and checked with each other, any disagreements were discussed with the third researcher (LC). We extracted the sample size, intervention, and control group characteristics, as well as the necessary raw data that were computed for the effect size (Hedges' g), such as mean, median, standard deviation, and standard error of pre- and post-cognitive outcomes. For median, range, and sample size, we used Hozo's method to transform the median and range to mean and standard deviation (Hozo et al., 2005). If the study was relevant to our analysis but the data necessary to calculate the effect sizes were missing, the authors were contacted via email to obtain the relevant data.

### 2.4 Outcome measures

Based on published reviews and factor analyses (Miyake et al., 2000; Friedman and Miyake, 2004; Rieker et al., 2022), the cognitive outcomes included objectively assessed cognitive domains of global cognition, executive control, memory, attention, processing speed, and verbal abilities. The results of cognitive screening tools, such as MoCA and MMSE, are classified as global cognition. The classification of executive functions included tests that measured working memory, inhibition, and cognitive flexibility. Memory included short- and long-term memory measurements. Attention included divided, selective, and sustained attention measures. Processing speed included tests that measured reaction times. Verbal fluency included assessments of verbal, categorical, and phonological fluency.

### 2.5 Risk of bias

Two authors (YQ and AS) evaluated the risk of bias independently in qualified studies using the Cochrane risk of bias in randomized trials tool (ROB-2), which includes five domains (1) Risk of bias arising from the randomization process; (2) Risk of bias due to deviations from the intended interventions; (3) Missing outcome data; (4) Risk of bias in measurement of the outcome; (5) Risk of bias in selection of the reported result. Disagreement was resolved through consultation between authors.

### 2.6 Statistical analyses

Raw data (sample size, mean, and standard deviation) of post-intervention outcomes across studies were collected and organized in an Excel spreadsheet, exported as a CSV format file, and then imported into R. All outcome measurements were treated as continuous variables. The meta-analysis was conducted in R Statistical Software (v4.4.1) with R packages meta (Balduzzi et al., 2019), metafor (Viechtbauer, 2010), dmetar (Harrer et al., 2019), clubSandwich (Pustejovsky, 2024), and esc (Lüdecke, 2019). A random-effects model was used due to anticipated variability between studies and corrected standardized mean difference (Hedges' g) and 95% confidence intervals (CI) were calculated to compare post-intervention outcomes between the exergaming and control groups. When multiple outcomes within the same cognitive domain were reported by a single study, effect sizes were aggregated at the study level using inverse-variance weighting to maintain statistical independence.

Heterogeneity was assessed using the *I*^2^ statistic and Cochran's Q test (χ^2^ *p*-value). The *I*^2^ statistic quantifies the percentage of variability in effect sizes attributable to true between-study heterogeneity rather than sampling error, with values of 25%, 50%, and 75% indicating low, moderate, and substantial heterogeneity, respectively (Higgins et al., 2003). A Cochran's Q *p*-value of less than 0.05 indicates significant heterogeneity. Restricted maximum likelihood estimation (REML) was used to estimate the between-study variance τ^2^ (Viechtbauer, 2005). Knapp-Hartung adjustments (Knapp and Hartung, 2003) were applied to calculate confidence intervals around pooled effects, which provides more conservative estimates particularly when the number of studies is small. The resulting t-statistic and associated p-value test the null hypothesis that the true pooled effect equals zero. For cognitive domains with only one study (k = 1), heterogeneity statistics could not be calculated, and the reported effect represents a single study finding rather than a meta-analytic estimate.

Between-study heterogeneity can be caused by one or more studies with extreme effect sizes, which may distort the pooled effect estimate of the meta-analysis (Harrer et al., 2021). Thus, outlier and influential case analyses were performed to identify studies or data points that have a strong influence on overall effect sizes. The function find.outlier in R package dmetar was applied to an object input created by an R meta package meta-analysis function. Baujat charts, the leave-one-out method, and funnel plots were used for outlier and influential case analysis; the leave-one-out method combined with forest plots provided recalculated pooled effects with one study omitted each time. Furthermore, funnel plots were used to visualize the symmetrical pattern of publications, with Egger's regression test (Egger et al., 1997) conducted to assess potential publication bias.

### 2.7 Subgroups analyses

Subgroup analyses were performed separately for cognitive domains (attention, executive function, global cognition, memory, verbal ability, and processing speed), population types (cognitively healthy older adults, people with dementia, and mild cognitive impairment), and exercise modes (aerobic, non-aerobic, and mixed), with appropriate aggregation applied to maintain one effect size per study within each subgroup category. All statistical tests used a significance level of *p* < 0.05.

## 3 Results

### 3.1 Database search

**Figure 1:**
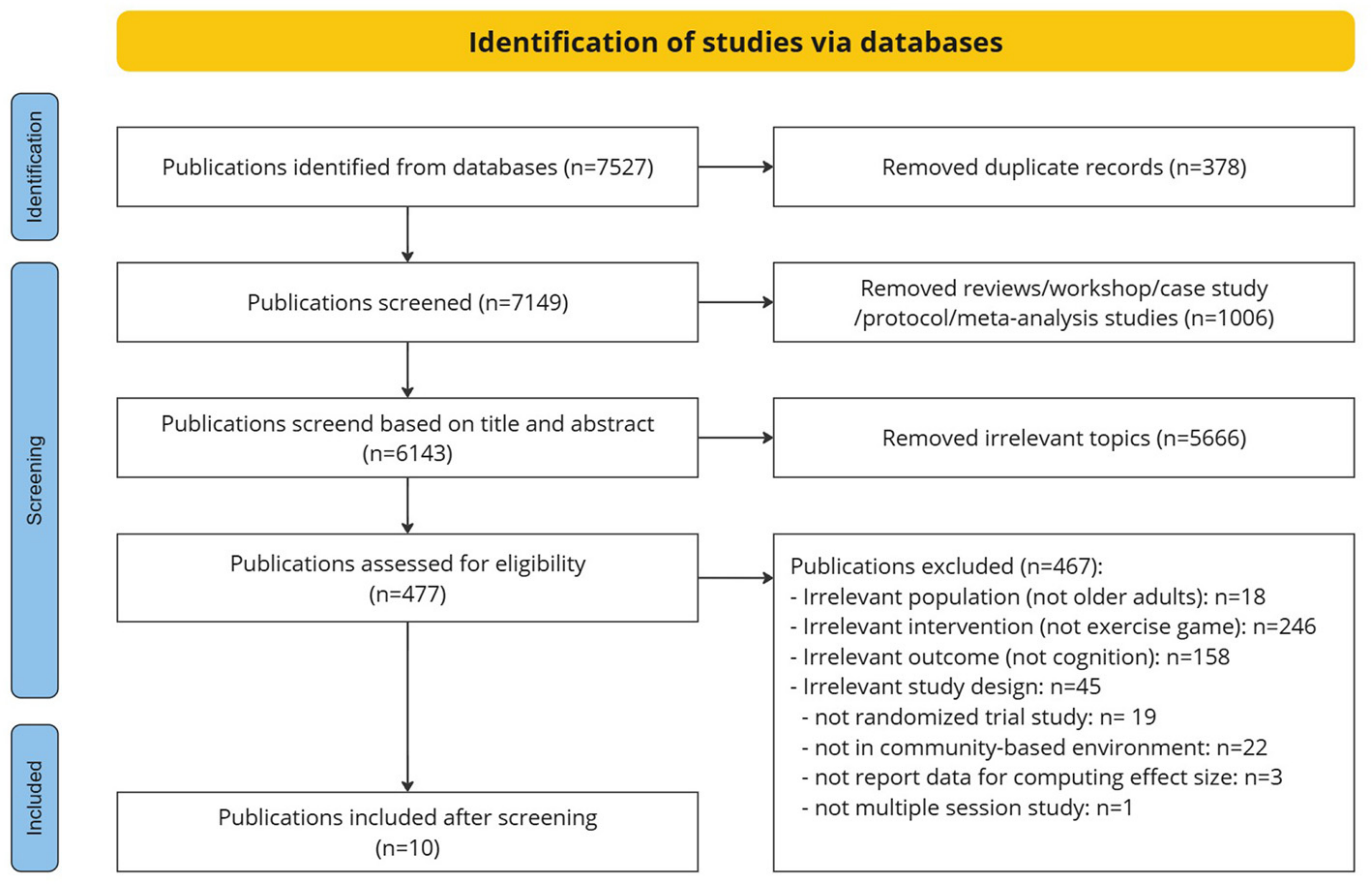
Preferred reporting items for systematic reviews and meta-analyses (PRISMA) flow diagram.

A total of 7,257 articles were found in the databases after we applied search terms (Figure 1). After removing duplications and non-intervention studies, such as reviews, workshops, case studies, and protocols, 6143 articles were left for screening. Of these, 5,666 articles were irrelevant to our interests based on the initial review of the title and abstract, and the irrelevant research include the ones focused on application, device development, or studies in different fields (e.g., K12 education, economics or sports, etc). Full text screening was performed with the remaining 477 studies, we screened out further studies due to irrelevant study design (mean age < 60, non-community-based environment, no randomized controlled trial, and not multiple session training). Three additional studies were excluded from the meta-analysis due to the need to report necessary data to compute the effect sizes. In total, our selection criteria left 10 studies for this meta-analysis (Stanmore et al., 2019; Martel et al., 2018; Gouveia et al., 2021; Gschwind et al., 2015b,a; Karssemeijer et al., 2019; Wu et al., 2023; Liao et al., 2021, 2020; Van Santen et al., 2020).

A total of 690 (EXG = 350, Control = 340) older adults participated in these 10 studies (Table 1). The mean age of the exercise game intervention group is 74.11 (SD = 21.29), and the mean age of the control group is 74.71 (SD = 21.19). One study does not report the exact mean age for each group but only a general range (e.g., 61–66, 71–75) so this study is excluded from calculating the overall mean age. Among all participants, 80 participants have mild cognitive impairment (MCI), 192 participants are people with dementia (PwD), and 418 participants are cognitively healthy older adults. Of all studies, only three studies include a follow-up assessment. Thus, the post-follow-up analysis is not included in this meta-analysis.

**Table 1:** Summary of studies.

References	Sample size	Mean Age (SD)	Pop.	#Sess.	Dur. (wks)	Sess./wk	Min/wk	Exercise game type	Exercise game intervention	Control	Cognitive measurements
Gschwind et al. (2015a)	100 EI:39 (f = 27) CT:61 (f = 40)	EI:82.5 (7) CT:80.2 (6.5)	Hlth.	48	16	3	20	Aero.	Musical stepping on mat	Health edu. material	Executive: Stroop; Memory: Digit span backwards
Gschwind et al. (2015b)	153 EI:78 (f = 43) CT:75 (f = 50)	EI:74.7 (6.7) CT:74.7 (6)	Hlth.	48	16	3	55-60	Non-aero.	Balance exercise games	Health edu. material	Executive: TMT-B, Stroop; Memory: Digit span; Attention: ANT
Martel et al. (2018)	28 EI:16 (f = 12) CT:12 (f = 9)	EI:74.9(7.1) CT:72.7(6.5)	Hlth.	24	12	2	55	Comb.	Home-based exercise games	Non-active control	Global: MoCA
Karssemeijer et al. (2019)	77 exp:38 (f = 18) cont:39 (f = 17)	EI:79 (6.9) CT:79.8 (6.5)	PwD	36	12	3	30-50	Aero.	Cogn.-aerobic cycling	Relaxation & flexibility	Executive: TMT-B, Rule Shift, Stroop; Processing: RT; Attention: TMT-A; Verbal: Letter Fluency; Memory: Location, Digit/Spatial Span
Stanmore et al. (2017)	106 EI:56 (f = 45) CT:50 (f = 38)	EI:77.9 (8.9) CT:77.8 (10.2)	Hlth.	36	12	3	33	Non-aero.	Otago w/ games	OTAGO exercises	Executive: ACE III
Van Santen et al. (2020)	91 EI:52 (f = 36) CT:39 (f = 16)	EI:79.0 (6) CT:79 (7)	PwD	48	24	2	150	Aero.	Interactive cycling	Regular activities	Global: MMSE; Executive: TMT-B; Attention: TMT-A
Gouveia et al. (2021)	31 EI:15 (f = 12) CT:16 (f = 10)	EI:67.6 (5) CT:69.1 (4.2)	Hlth.	24	12	2	45	Comb.	Five custom exercise games	Group trad. training	Global: COGTEL; Executive: Inductive reasoning; Memory: LT/ST verbal; Verbal: Fluency
Liao et al. (2020)	34 EI:18 (f = 11) CT:16 (f = 12)	EI:75.5 (5.2) CT:73.1 (6.8)	MCI	36	12	3	60	Comb.	VR-based phys.-cogn. training	Combined phys. & cogn. training	Global: MoCA, EXIT-25; Memory: CCVLT
Liao et al. (2021)	46 EI:25 (f = 16) CT:21 (f = 15)	EI:79.6 (9) CT:83.8 (5.1)	MCI	36	12	3	60	Comb.	Aerobic, resistance, Tai Chi & balance on Kinect	Combined phys. exercise	Global: MoCA, EXIT-25; Memory: CCVLT; Executive: TMT-B, Stroop, N-back 1-2
Wu et al. (2023)	24 EI=13 (f = 8) CT=11 (f = 8)	EI:78.8 (4.8) CT:81.2 (4.4)	PwD	36	12	3	30-50	Aero.	ExerHeart w/ video game	Indoor cycling	Executive: Flanker

EI, Exergaming group; CT, Control group; Pop., Population (Hlth., Healthy; PwD, People with Dementia); Type = exercise game Type (Aero., Aerobic; Comb., Combined; Non-aero., Non-aerobic); RT, Reaction Time; LT, Long-term; ST, Short-term. TMT-A/TMT-B, Trial Making Test; ANT, Attention Network Test; MoCA, Montreal Cognitive Assessment; MMSE, Mini-Mental State Examination; ACE III, Addenbrooke's Cognitive Examination; CCVLT, Chinese Version Verbal Learning Test; COGTEL, Cognitive Telephone Screening Instrument; EXIT-25, Executive Interview.

The intervention training ranges from 12 to 24 weeks; seven studies conducted a 12-week intervention, two studies did a 16-week intervention, and one did a 24-week intervention. In terms of the frequency of the sessions, all studies chose to do either two sessions per week or three sessions per week. The duration of each session varies substantially among the studies, ranging from 20 to 150 minutes per session.

### 3.2 Risk of bias

**Figure 2:**
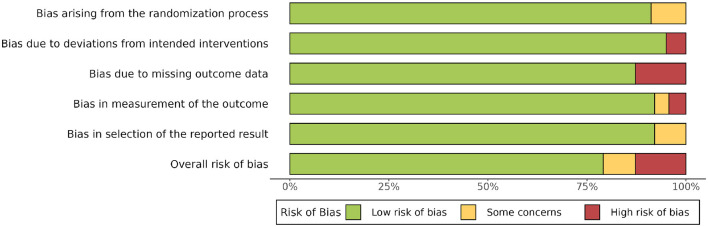
The overall risk of bias among involved studies.

Among the 10 studies (Table 2), six are categorized as “low risk,” two studies are at “some concerns,” and two studies are at “high risk.” The weighted bias figure is shown below (Figure 2). For all five bias domains, the overall bias level is low in our meta-analysis.

**Table 2:** The risk of bias evaluation on each study by risk domains.

References	D1	D2	D3	D4	D5	Overall	Weight
Gschwind et al. (2015a)	Low	Low	Low	Low	Low	Low	23.5998
Gschwind et al. (2015b)	Low	Low	Low	Low	Low	Low	38.0689
Martel et al. (2018)	Low	Low	Low	Some concerns	Some concerns	Some concerns	6.0475
Karssemeijer et al. (2019)	Low	Low	Low	Low	Low	Low	16.5734
Stanmore et al. (2017)	Low	Low	Low	Low	Low	Low	22.8772
Van Santen et al. (2020)	Low	Low	Low	Low	Low	Low	19.7599
Gouveia et al. (2021)	Some concerns	Low	Low	Low	Low	Some concerns	7.5124
Liao et al. (2020)	Low	High	High	Low	Low	High	8.4421
Liao et al. (2021)	Low	Low	Low	Low	Low	Low	11.1865
Wu et al. (2023)	Low	Low	High	Low	Low	High	5.7985

D1 - Risk of bias arising from the randomization process; D2 - Risk of bias due to deviations from the intended interventions; D3 - Missing outcome data; D4 - Risk of bias in the measurement of the outcome; D5 - Risk of bias in the selection of the reported result.

### 3.3 Exergame design and implementation characteristics

The analysis of exergame features across the included studies (*n* = 10) revealed substantial heterogeneity in the completeness and depth of game design reporting (see Supplementary Tables S1, S2). Overall, the description of exergame characteristics was minimal across most studies, with reporting predominantly focused on movement mechanics and input-output mapping rather than comprehensive game design elements.

The majority of studies (8/10, 80%) primarily described exergames through their physical movement requirements and corresponding on-screen actions. Critical game design components were rarely reported across the reviewed studies. No studies explicitly described graphical style elements such as 2D versus 3D rendering, realistic versus stylized art direction, color palettes, or visual themes. Core game mechanics including scoring systems, difficulty progression algorithms, level structures, win or loss conditions, or reward mechanisms were largely unreported. Elements known to enhance player engagement—including competition modes (individual vs. multiplayer), progress tracking systems, achievement systems, narrative elements, or personalization options—were absent from most descriptions. While some studies mentioned “visual and auditory feedback” (Liao et al., 2020, 2021; Martel et al., 2018), the specific nature, timing, or purpose of this feedback remained unspecified. Only two studies provided slightly more comprehensive descriptions (Gschwind et al., 2015a; Gouveia et al., 2021)

### 3.4 Feasbility and safety

Attrition rates across the included studies ranged from 10.5% to 50% (median = 17.65%), see Supplementary Tables S1, S2, with most withdrawals attributed to personal health issues unrelated to the intervention, changes in living arrangements, or death, while a minority of cases cited low motivation or technical difficulties as reasons for discontinuation. Notably, studies explicitly reporting motivation-related attrition included (Liao et al., 2021), where two participants found the program “not attractive to them,” (Van Santen et al., 2020), where seven participants wanted to stop, and (Gschwind et al. 2015a), which reported system failures and technical issues as barriers. All included studies reported no adverse events related to the exergaming interventions, with (Gschwind et al. 2015a) and (Gouveia et al. 2021) specifically noting no falls or injuries associated with gameplay. These findings suggest that home-based and community-based exergaming interventions are both feasible and safe for older adults; however, enhancing game playability to increase enjoyment and simplifying technical setup to improve accessibility remain critical considerations for sustaining participant engagement and minimizing intervention-related attrition.

### 3.5 Effect of exercise game on cognition

**Figure 3:**
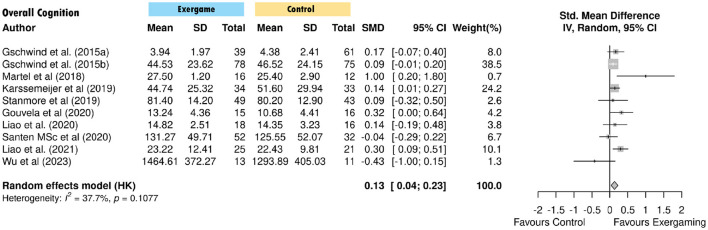
Pooled effect of exercise game in overall cognition. SD, Standard Deviation; SMD, Standardized Mean Difference; IV, Inverse Variance; CI, Confidence Interval.

**Figure 4:**
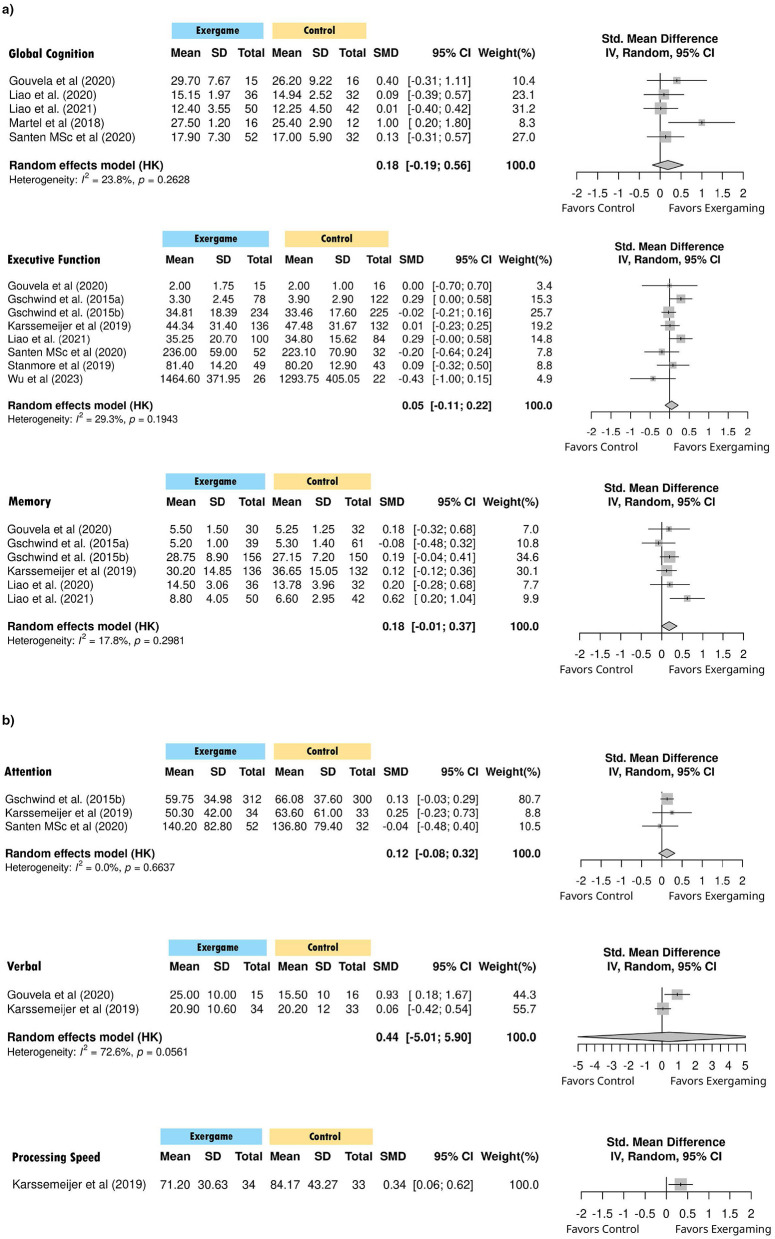
**(a)** Forest plot of standardized mean differences in global cognition, executive function and memory. **(b)** Forest plot of standardized mean differences in attention, verbal fluency, and processing speed.

Exercise game interventions produced a small but significant overall effect on cognitive function (g = 0.135, 95% CI [0.037, 0.229], *p* = 0.012, *I*^2^ = 37.7%) (Table 3, Figure 3). Domain-specific analyses revealed non-significant effects for global cognition (g = 0.185, *p* = 0.240), executive function (g = 0.054, *p* = 0.473), memory (g = 0.179, *p* = 0.060), and attention (g = 0.124, *p* = 0.118), though memory approached statistical significance (Figure 4a). Processing speed showed a significant effect based on one study (g = 0.337, *p* = 0.018), while verbal function results were inconclusive due to high heterogeneity (*I*^2^ = 72.6%) and imprecise estimates (Figure 4b).

**Table 3:** Effect of exercise game in comparison to control group on cognition.

Characteristics	No. of studies	Summary effect		Null Hypothesis		Heterogeneity	
		**Hedges' g**	**95% CI**	**t**	***p*** **-value**	*I* ^2^ **(%)**	**Q**	***p*** **-value**
Cognition								
Overall	10	0.135	[0.037; 0.229]	3.12	0.012^*^	37.7	14.44	0.1077
Global	5	0.185	[–0.187; 0.557]	1.38	0.240	23.8	5.25	0.2628
Executive function	8	0.054	[–0.114; 0.221]	0.76	0.473	29.3	9.90	0.1943
Memory	6	0.179	[–0.011; 0.371]	2.42	0.060	17.8	6.08	0.2981
Attention	3	0.124	[–0.077; 0.324]	2.65	0.118	0	0.82	0.6637
Processing speed	1	0.337	[0.059; 0.616]	2.37	0.018^*^	-	3.65	-
Verbal	2	0.444	[–5.009; 5.897]	1.03	0.489	72.6	0.00	0.0561
**Sub.population**							3.21	0.2007
Cognitive healthy	5	0.149	[–0.045; 0.343]	2.13	0.101	39.4	6.60	0.1583
MCI	2	0.256	[–0.621; 1.133]	3.71	0.168	0	0.59	0.4433
PwD	3	0.005	[–0.558; 0.567]	0.04	0.975	59.5	4.60	0.1005
**Sub.exercise game**							2.91	0.2332
Aerobic	5	0.119	[–0.062; 0.300]	1.83	0.142	40.5	6.72	0.1515
Combined	3	0.291	[–0.229; 0.811]	2.41	0.138	47.1	3.78	0.1513
Non-aerobic	2	0.091	[0.078; 0.104]	90.58	0.007^**^	0	0	0.9846

MCI, People with mild cognitive impairment; PwD, People with dementia.

^*^indicates *p* < 0.05;

^**^indicates *p* < 0.01.

### 3.6 Subgroup: exercise game types

**Figure 5:**
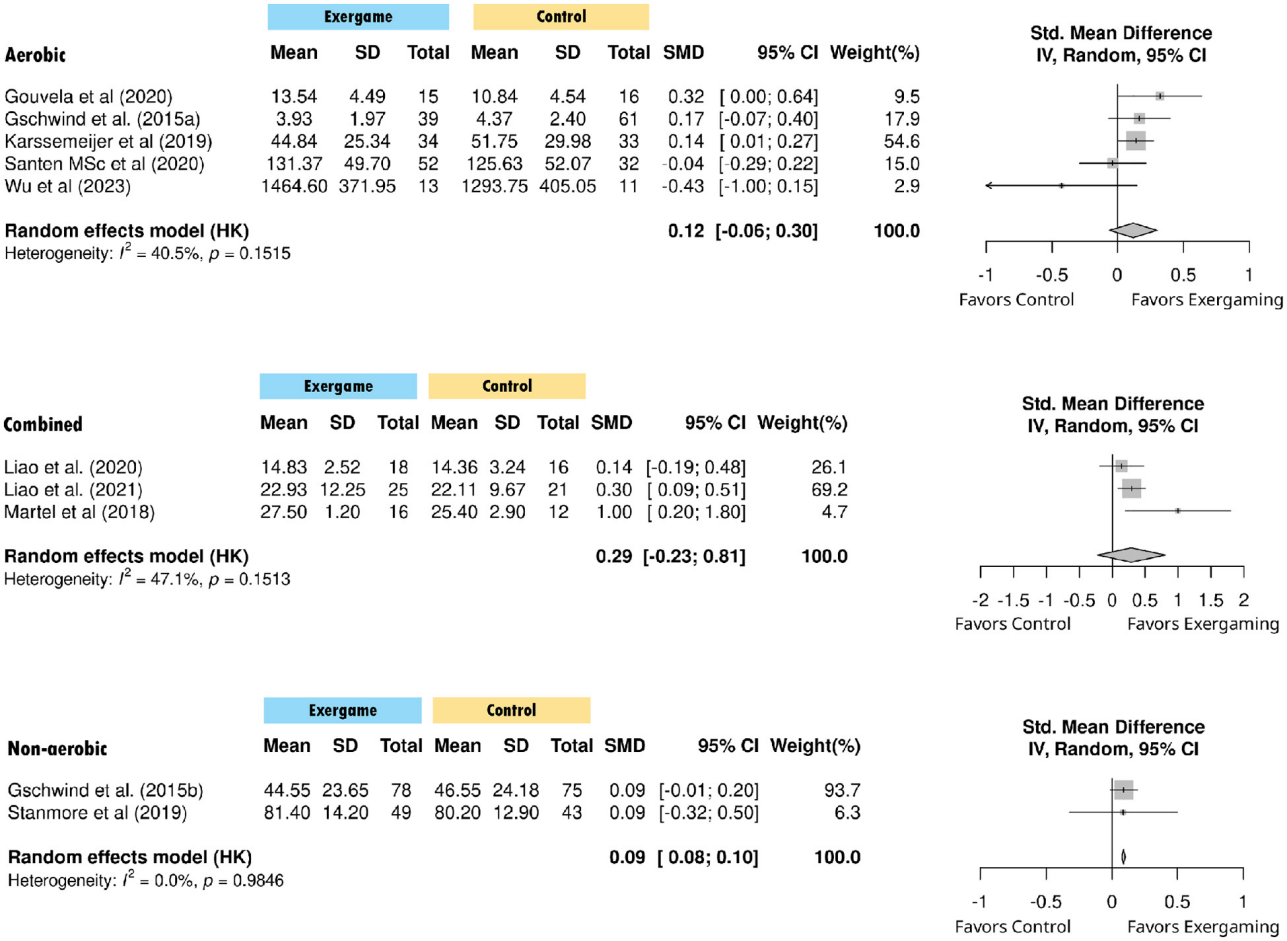
Forest plot of standardized mean differences in cognitive outcomes by exercise game type.

Aerobic exergaming yielded a small positive effect that approached but did not reach statistical significance (g = 0.119, *p* = 0.142), with moderate heterogeneity (*I*^2^ = 40.5%, Q = 6.72, *p* = 0.152). Combined exercise programs demonstrated a small-to-moderate effects (g = 0.291, *p* = 0.138), with moderate heterogeneity (*I*^2^ = 47.1%, Q = 3.78, *p* = 0.151). Non-aerobic exergaming showed a negligible but significant effect (g = 0.091, *p* = 0.007^**^), without evidence of heterogeneity (*I*^2^ = 0%, Q = 0, *p* = 0.985). The test for subgroup difference was not statistically significant (Q = 2.91, *p* = 0.233), suggesting that intervention effects did not vary significantly by exercise modality (Figure 5).

### 3.7 Subgroup: population types

**Figure 6:**
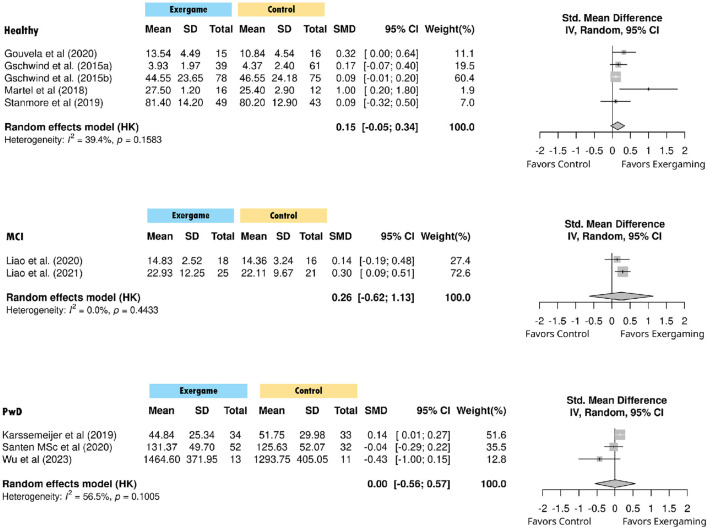
Forest plot of standardized mean differences in cognitive outcomes by population type.

Subgroup analyses by population type included 10 studies across three categories. Cognitively healthy older adults showed a small positive effect that did not reach statistical significance (g = 0.149, *p* = 0.101), with moderate but not significant heterogeneity (*I*^2^ = 39.4%, Q = 6.60, *p* = 0.158). Individuals with mild cognitive impairment (MCI) demonstrated a small-to-moderate effect that was not statistically significant (g = 0.256, *p* = 0.168), with no evidence of heterogeneity (*I*^2^ = 0%, Q = 0.59, *p* = 0.443). People with dementia (PwD) (k = 3) showed virtually no effect (g = 0.005, *p* = 0.975), with substantial heterogeneity (*I*^2^ = 59.5%, Q = 4.60, *p* = 0.101). The test for subgroup differences was not statistically significant (Q = 3.21, *p* = 0.201) (Figure 6).

## 4 Discussion

This meta-analysis investigated the effects of exercise game interventions on the cognitive functions of older adults who live in communities with or without assisted facilities. It includes 10 eligible studies that explored the differential effects of exercise game interventions versus control groups. The included studies varied in study design, game platforms, and the specific exercise games employed. The control groups also differed, encompassing active, non-active, and traditional exercise interventions.

### 4.1 The effects of exercise games on cognition

In contrast to traditional cognitive training, which often exhibits limited transfer effects (Owen et al., 2010; Peckham, 2021), our findings demonstrate that exercise games exert a broader influence on cognitive function among community-dwelling older adults. Exercise games demonstrated preliminary evidence of improvements on memory and processing speed, and these results align with previous research highlighting the positive impact of exercise games on short-term memory (Roig et al., 2013) and processing speed (Moret et al., 2022). It could be that the inherent cognitive demands of exercise games may stimulate neural pathways in a manner that is distinct from traditional physical exercise (Eggenberger et al., 2016; Liao et al., 2019), potentially leading to unique neurophysiological adaptations and enhanced cognitive outcomes. However, global cognition, executive function, attention, and verbal fluency did not display significant improvements. It is worth mentioning that the relatively small number of studies focusing on processing speed and verbal fluency precluded definitive conclusions regarding the impact of exercise games on these domains.

In addition, the available outcome assessments only tap a small part of the broader constructs of cognitive functions, such as attention and verbal fluency. This led to another issue, for which accurate assessment of cognition remains a critical challenge in a community-based environment. Also, the inconsistent application of cognitive assessment tools, as exemplified by the varied categorization of the TMT-A task, can confound interpretation. Moreover, the use of screening tools such as the MoCA and MMSE as outcome assessments creates limitations for our interpretation of the findings, especially for the study that used screening tools as the only cognitive measurement. This point underscores the need for more comprehensive cognitive evaluations with detailed cognitive profiles to better characterize the outcomes. Identifying readily accessible yet reliable cognitive assessment tools for community-based research remains an ongoing challenge.

### 4.2 The effects of exercise games on non-clinical and clinical populations

Our meta-analysis examined the cognitive effects of exercise games across cognitively healthy older adults, individuals with mild cognitive impairment (MCI), and those with dementia (PwD). However, the evidence base for clinical populations remains severely limited and does not support definitive conclusions. With only two studies examining MCI participants and three studies examining dementia, none of these clinical subgroups demonstrated statistically significant effects (MCI: g = 0.256, *p* = 0.168; PwD: g = 0.005, *p* = 0.975). While the MCI subgroup showed a numerically larger effect size compared to healthy controls, this difference was not statistically significant and is based on an insufficient number of studies to draw meaningful inferences. Given the substantial heterogeneity in dementia studies (*I*^2^ = 59.5%) and the non-significant test for subgroup differences (Q = 3.21, *p* = 0.201), we cannot determine whether exercise games produce differential effects across population types. Previous hypotheses, such as the “compensation hypothesis” suggesting greater neuroplasticity potential in individuals with lower baseline cognitive function (Sanders et al., 2019; Wu et al., 2019), or neurobiological constraints in advanced cognitive decline (Arnold et al., 2013; Buschert et al., 2011), remain speculative in the context of exergaming interventions due to insufficient empirical evidence. The critical gap in the current literature is the lack of adequately powered randomized controlled trials specifically enrolling individuals with MCI and dementia (Voinescu et al., 2021). Future research must prioritize well-designed studies with sufficient sample sizes in these clinical populations, employ comprehensive and standardized cognitive assessment batteries, systematically document intervention fidelity and adherence, and report feasibility and safety outcomes to establish whether exergaming interventions offer meaningful cognitive benefits for individuals with cognitive impairment in community settings.

### 4.3 The effects of exercise games by exercise type

Our subgroup analysis revealed that the cognitive benefits derived from exercise game interventions vary according to exercise type. Exercise games that integrated aerobic, resistance, and balance components yielded the most pronounced cognitive benefits. Aerobic exercise game intervention alone shows a small positive impact. In contrast, non-aerobic exercise alone did not produce substantial cognitive improvements.

Extensive research supports the positive influence of aerobic exercise and resistance training on cognition (Colcombe et al., 2006; Hillman et al., 2008; Erickson et al., 2009; Huang et al., 2022; Li et al., 2018). However, the impact of non-aerobic training, such as resistance exercises, on cognitive function is less well-established. Some studies have reported benefits for specific cognitive domains, but the evidence is inconsistent (Rogge et al., 2017; Dorner et al., 2007). Our findings align with a recent review suggesting that multicomponent exercise regimens, incorporating aerobic elements, are essential for optimizing cognitive outcomes in older adults (Yan et al., 2023). While the optimal exercise modality for enhancing cognitive function in older adults remains a subject of ongoing debate, our results underscore the importance of integrating aerobic components into exercise game interventions to maximize cognitive benefits. There is a new direction for developing games that blend cognitive and physical activity to promote the cognitive health of older adults as some current studies show (Anguera et al., 2022; Qian et al., 2024).

### 4.4 Implications for stakeholders

Exercise games represent a promising intervention modality for mitigating age-related physical and cognitive decline in older adults. A key advantage of exercise games lies in their scalability and accessibility, making them uniquely well-suited for home- and community-based interventions. However, our findings reveal a notable discrepancy: while laboratory-based studies have demonstrated significant moderate to large effect sizes for exercise game interventions (Yen and Chiu, 2021; Stanmore et al., 2017; Peng et al., 2024), community-based implementations show considerably attenuated clinical effects.

Understanding the factors contributing to this reduced efficacy in community settings is critical for optimizing the translation of exercise game interventions from controlled research environments to real-world applications. Several hypotheses warrant consideration. First, the mode of delivery and implementation fidelity may differ substantially between laboratory and community contexts, potentially affecting adherence, intensity, and overall intervention quality. Second, the characteristics of the exercise games themselves may play a pivotal role in determining outcomes. However, the majority of studies in this review focus primarily on reporting physical movement requirements while neglecting core game design elements—such as progress tracking, timing mechanics, competition, and scoring systems—that are integral to sustaining cognitive engagement. This reporting gap limits our understanding of why certain games may be more effective for specific cognitive domains than others. Moreover, without sufficient access to detailed game design specifications, important questions remain unresolved: Do specific cognitive elements embedded within games differentially affect particular cognitive functions? To what extent are observed cognitive benefits attributable to the physical exercise component versus the cognitive demands inherent in gameplay? Addressing these questions is essential for disentangling the mechanisms of action underlying exergame efficacy and for informing the evidence-based design of future interventions.

Notably, our meta-analysis reveals an important distinction from previous reviews examining commercial exergaming platforms (e.g., *Nintendo Wii, VR Dance*, and *Xbox Kinect Sports*) (Yen and Chiu, 2021; Stanmore et al., 2017; Peng et al., 2024) for older adults. The majority of studies included in the present analysis employed researcher-developed games rather than commercially available products. This raises a critical question: do the underlying game mechanics and design principles influence cognitive outcomes, even among interventions broadly classified as exercise games or movement-based games? The heterogeneity in game design, interaction paradigms, and cognitive engagement strategies may contribute meaningfully to the observed variability in treatment effects.

Given these considerations, the following sections discuss the implications of our findings for two key stakeholder groups: (1) designers and developers creating future exercise game interventions, and (2) researchers conducting investigations with exercise game platforms in community settings.

#### 4.4.1 Implications for exercise game designers and developers

Our findings have several important implications for exercise game developers and designers. First, prioritizing multicomponent exercise is crucial, as our results strongly suggest that incorporating both aerobic and non-aerobic elements into exercise games is essential for maximizing cognitive benefits. Developers should focus on creating games that elevate heart rate, provide resistance exercises, and engage players cognitively, as resistance activities not only enhance cognitive function in older adults (Li et al., 2018; Huang et al., 2022) but also help maintain and build muscle mass, supporting motor functions (Mayer et al., 2011) and facilitating participation in repetitive exercises, such as aerobics. Second, while exercise games have shown broad cognitive benefits, targeting specific cognitive domains—particularly memory and processing speed—could be particularly advantageous, and designing games with challenges that explicitly address these domains might enhance their impact. Third, recognizing the differential effects of exercise games on different populations, developers could create tailored experiences for healthy older adults, individuals with MCI, and those with dementia to optimize outcomes for each group. Finally, making in-game data collection available to researchers through special access would enable researchers to have a better understanding of individual progress within exercise games and how that relates to cognitive outcomes, thereby advancing both practical application and scientific knowledge in this field.

#### 4.4.2 Implications for researchers

Our findings highlight several important directions for future research. First, future research should prioritize studies investigating the effects of aerobic and multicomponent exercise games on cognition in older adults, as these intervention types show the most promise. Second, to better understand the underlying mechanisms of cognitive improvement, research should delve deeper into cognitive mechanisms by exploring the neurophysiological changes associated with exercise game interventions (e.g., using techniques such as EEG, ERPs, MRI, and fMRI), and future research should also explore whether specific types of memory, such as working memory, procedural memory, declarative memory, and long-term memory, show the more substantial benefits of exercise games. Third, addressing assessment limitations is essential for advancing the field; developing and validating more sensitive and specific cognitive assessment tools for use in community-based settings is critical, and additionally, researchers need tools that can deploy the assessments, ideally, into the game platform by collaborating with game designers and developers to better understand what happens during gameplay and how it is related to cognitive outcomes. Fourth, longitudinal studies are needed to investigate long-term effects by examining the long-term cognitive benefits of exercise games and to determine if these effects persist after completing the intervention. Finally, standardizing exercise game reporting is crucial—researchers should be encouraged to provide detailed descriptions of exercise games, including the average heart rate range during exercise, game mechanics, difficulty levels, and feedback mechanisms. It is important to observe whether different modes of exercise games (e.g., target-hitting, strategy, and narrative) may yield varying cognitive benefits, with each type potentially showing the largest gains in its corresponding cognitive domain (e.g., attention, reasoning, and verbal skills respectively), as this standardization will facilitate the categorization of exercise types, comparisons, and meta-analyses.

## 5 Limitations

This meta-analysis has several limitations related to both the included evidence and the review methodology. First, the small number of eligible studies (*n* = 10) and variability in sample sizes across these studies limited the statistical power and precision of pooled estimates; this also constrains the reliability of publication bias assessments, as funnel plot-based methods and Egger's regression are known to be underpowered when fewer than 10–15 studies are available, increasing the risk of false-positive and false-negative results. Therefore, publication bias assessments should be interpreted with considerable caution, and the potential for unpublished null findings to alter our conclusions cannot be ruled out. Additionally, our ≥12-week intervention criterion, while grounded in dose-response literature suggesting that longer interventions produce more robust cognitive benefits (Sanders et al., 2019), may have excluded shorter yet potentially informative studies. Some cognitive and physical training programs of 6-8 weeks duration have demonstrated measurable cognitive effects in specific domains (Smith et al., 2009), and excluding these studies may have limited our ability to examine dose-response relationships and reduced the overall evidence base.

Consequently, specific cognitive domains were underrepresented, with processing speed and verbal fluency each having insufficient studies (k = 1–2) to conduct meaningful meta-analytic synthesis or assess heterogeneity. The preponderance of studies focused on cognitively healthy older adults, limiting the generalizability of findings to clinical populations, particularly people with dementia where only three studies were available.

Second, substantial methodological heterogeneity existed across the included studies. Cognitive outcomes were measured using diverse assessment instruments with varying psychometric properties, making direct comparisons challenging. Exergaming interventions varied considerably in game type (e.g., dancing, sports, and balance games), exercise intensity, session duration, and total intervention length, which may have contributed to differential effects that could not be fully disentangled through subgroup analyses due to limited study numbers. Additionally, control group conditions were inconsistent, ranging from passive waitlist controls to active control interventions, potentially influencing the magnitude of observed effects.

Despite these limitations, the generally low-to-moderate heterogeneity between studies (*I*^2^ = 0%–40.5% for most analyses) and low weighted risk of bias across methodological domains suggest reasonable consistency in the observed effects. Future research would benefit from standardized cognitive assessment batteries, detailed reporting of intervention parameters, larger and more diverse samples including underrepresented clinical populations, and exploration of dose-response relationships to establish optimal exergaming protocols for cognitive enhancement in older adults.

## 6 Conclusion

This systematic review and meta-analysis examined the effectiveness of exergaming interventions on cognitive outcomes in community-dwelling older adults, including those with mild cognitive impairment and dementia. The findings demonstrate that exergaming produces small but statistically significant improvements in overall cognitive function, though the evidence base for clinical populations remains limited and inconclusive, with only two studies examining MCI and three examining dementia, neither showing statistically significant effects. Multicomponent exercise programs incorporating aerobic elements showed numerically larger, though non-significant, effects compared to other modalities, suggesting that exercise type may influence outcomes, though more research is needed to confirm these patterns. These results provide preliminary evidence supporting the potential of exergaming as a modest, accessible supplementary intervention for cognitive health in community settings. However, the small effect sizes and limited evidence in clinical populations indicate that exergaming should be considered as one component of a comprehensive approach to cognitive health rather than a standalone intervention. Further research with larger samples, standardized protocols, and systematic safety reporting is needed to establish optimal implementation strategies and determine long-term efficacy.

## Data Availability

The original contributions presented in the study are included in the article/Supplementary material, further inquiries can be directed to the corresponding author.

## References

[B1] Anguera J. A. Volponi J. J. Simon A. J. Gallen C. L. Rolle C. E. Anguera-Singla R. . (2022). Integrated cognitive and physical fitness training enhances attention abilities in older adults. NPJ Aging 8:12. doi: 10.1038/s41514-022-00093-y 36042247 PMC9427998

[B2] Arnold S. E. Louneva N. Cao K. Wang L.-S. Han L.-Y. Wolk D. A. . (2013). Cellular, synaptic, and biochemical features of resilient cognition in Alzheimer's disease. Neurobiol. Aging 34, 157–168. doi: 10.1016/j.neurobiolaging.2012.03.004 22554416 PMC3478410

[B3] Balduzzi S. Rücker G. Schwarzer G. (2019). How to perform a meta-analysis with R: a practical tutorial. Evid. Based Mental Health 22, 153–160. doi: 10.1136/ebmental-2019-300117 31563865 PMC10231495

[B4] Buschert V. C. Friese U. Teipel S. J. Schneider P. Merensky W. Rujescu D. . (2011). Effects of a newly developed cognitive intervention in amnestic mild cognitive impairment and mild Alzheimer's disease: a pilot study. J. Alzheimer's Dis. 25, 679–694. doi: 10.3233/JAD-2011-100999 21483095

[B5] Chiao C.-Y. Wu H.-S. Hsiao C.-Y. (2015). Caregiver burden for informal caregivers of patients with dementia: a systematic review. Int. Nurs. Rev. 62, 340–350. doi: 10.1111/inr.12194 26058542

[B6] Colcombe S. J. Erickson K. I. Scalf P. E. Kim J. S. Prakash R. S. McAuley E. . (2006). Aerobic exercise training increases brain volume in aging humans. J. Gerontol. A Biol. Sci. Med. Sci. 61, 1166–1170. doi: 10.1093/gerona/61.11.1166 17167157

[B7] Craig T. V. Rhodes R. E. Sui W. (2024). Examining and comparing the energy expenditure of two modes of a virtual reality fitness game (supernatural): indirect calorimetry study. JMIR Serious Games 12:e53999. doi: 10.2196/53999 38833285 PMC11185914

[B8] Dedeyne L. Deschodt M. Verschueren S. Tournoy J. Gielen E. (2017). Effects of multi-domain interventions in (pre)frail elderly on frailty, functional, and cognitive status: a systematic review. Clin. Interv. Aging 12, 873–896. doi: 10.2147/CIA.S130794 28579766 PMC5448695

[B9] Dorner T. Kranz A. Zettl-Wiedner K. Ludwig C. Rieder A. Gisinger C. (2007). The effect of structured strength and balance training on cognitive function in frail, cognitive impaired elderly long-term care residents. Aging Clin. Exp. Res. 19, 400–405. doi: 10.1007/BF03324721 18007119

[B10] Eggenberger P. Wolf M. Schumann M. De Bruin E. D. (2016). Exergame and balance training modulate prefrontal brain activity during walking and enhance executive function in older adults. Front. Aging Neurosci. 8:66. doi: 10.3389/fnagi.2016.00066 27148041 PMC4828439

[B11] Egger M. Smith G. D. Schneider M. Minder C. (1997). Bias in meta-analysis detected by a simple, graphical test. BMJ 315, 629–634. doi: 10.1136/bmj.315.7109.629 9310563 PMC2127453

[B12] Erickson K. I. Hillman C. Stillman C. M. Ballard R. M. Bloodgood B. Conroy D. E. . (2019). Physical activity, cognition, and brain outcomes: a review of the 2018 physical activity guidelines. Med. Sci. Sports Exerc. 51, 1242–1251. doi: 10.1249/MSS.0000000000001936 31095081 PMC6527141

[B13] Erickson K. I. Prakash R. S. Voss M. W. Chaddock L. Hu L. Morris K. S. . (2009). Aerobic fitness is associated with hippocampal volume in elderly humans. Hippocampus 19, 1030–1039. doi: 10.1002/hipo.20547 19123237 PMC3072565

[B14] Friedman N. P. Miyake A. (2004). The relations among inhibition and interference control functions: a latent-variable analysis. J. Exper. Psychol. 133:101. doi: 10.1037/0096-3445.133.1.101 14979754

[B15] Gavelin H. M. Dong C. Minkov R. Bahar-Fuchs A. Ellis K. A. Lautenschlager N. T. . (2021). Combined physical and cognitive training for older adults with and without cognitive impairment: a systematic review and network meta-analysis of randomized controlled trials. Ageing Res. Rev. 66:101232. doi: 10.1016/j.arr.2020.101232 33249177

[B16] Ghai S. Ghai I. Effenberg A. O. (2017). Effects of dual tasks and dual-task training on postural stability: a systematic review and meta-analysis. Clin. Interv. Aging 12, 557–577. doi: 10.2147/CIA.S125201 28356727 PMC5367902

[B17] Gomez D. H. Bagley J. R. Bolter N. Kern M. Lee C. M. (2018). Metabolic cost and exercise intensity during active virtual reality gaming. Games Health J. 7, 310–316. doi: 10.1089/g4h.2018.0012 30325233

[B18] Gouveia l,. R. Smailagic A. Ihle A. Marques A. Gouveia B. R. Cameirão M. . (2021). The efficacy of a multicomponent functional fitness program based on exergaming on cognitive functioning of healthy older adults: a randomized controlled trial. J. Aging Phys. Activ. 29, 586–594. doi: 10.1123/japa.2020-0083 33361495

[B19] Gschwind Y. J. Eichberg S. Ejupi A. De Rosario H. Kroll M. Marston H. R. . (2015a). ICT-based system to predict and prevent falls (iStoppFalls): results from an international multicenter randomized controlled trial. Eur. Rev. Aging Phys. Activ. 12:10. doi: 10.1186/s11556-015-0155-6 26865874 PMC4748323

[B20] Gschwind Y. J. Schoene D. Lord S. R. Ejupi A. Valenzuela T. Aal K. . (2015b). The effect of sensor-based exercise at home on functional performance associated with fall risk in older people – a comparison of two exergame interventions. Eur. Rev. Aging Phys. Activ. 12:11. doi: 10.1186/s11556-015-0156-5 26865875 PMC4748327

[B21] Harrer M. Cuijpers P. A. F. T Ebert D. D. (2021). Doing Meta-Analysis With R: A Hands-On Guide. Boca Raton, FL and London: Chapman and Hall/CRC Press, 1st edition. doi: 10.1201/9781003107347

[B22] Harrer M. Cuijpers P. Furukawa T. Ebert D. D. (2019). dmetar: Companion R Package For The Guide 'Doing Meta-Analysis in R'. R package version 0.1.0.

[B23] Higgins J. P. Thompson S. G. Deeks J. J. Altman D. G. (2003). Measuring inconsistency in meta-analyses. BMJ 327, 557–560. doi: 10.1136/bmj.327.7414.557 12958120 PMC192859

[B24] Hill N. T. Mowszowski L. Naismith S. L. Chadwick V. L. Valenzuela M. Lampit A. (2017). Computerized cognitive training in older adults with mild cognitive impairment or dementia: a systematic review and meta-analysis. Am. J. Psychiatry 174, 329–340. doi: 10.1176/appi.ajp.2016.16030360 27838936

[B25] Hillman C. H. Erickson K. I. Kramer A. F. (2008). Be smart, exercise your heart: exercise effects on brain and cognition. Nat. Rev. Neurosci. 9, 58–65. doi: 10.1038/nrn2298 18094706

[B26] Howes S. C. Charles D. K. Marley J. Pedlow K. McDonough S. M. (2017). Gaming for health: systematic review and meta-analysis of the physical and cognitive effects of active computer gaming in older adults. Phys. Ther. 97, 1122–1137. doi: 10.1093/ptj/pzx088 29077911

[B27] Hozo S. P. Djulbegovic B. Hozo I. (2005). Estimating the mean and variance from the median, range, and the size of a sample. BMC Med. Res. Methodol. 5, 1–10. doi: 10.1186/1471-2288-5-13 15840177 PMC1097734

[B28] Huang X. Zhao X. Li B. Cai Y. Zhang S. Wan Q. . (2022). Comparative efficacy of various exercise interventions on cognitive function in patients with mild cognitive impairment or dementia: a systematic review and network meta-analysis. J. Sport Health Sci. 11, 212–223. doi: 10.1016/j.jshs.2021.05.003 34004389 PMC9068743

[B29] Kappen D. L. Nacke L. E. Gerling K. M. Tsotsos L. E. (2016). “Design strategies for gamified physical activity applications for older adults,” in 2016 49th Hawaii International Conference on System Sciences (HICSS) (New York, NY: Proceedings of the IEEE HICSS), 1309–1318. doi: 10.1109/HICSS.2016.166

[B30] Karssemeijer E. G. A. Aaronson J. A. Bossers W. J. R. Donders R. Olde Rikkert M. G. M. Kessels R. P. C. (2019). The quest for synergy between physical exercise and cognitive stimulation via exergaming in people with dementia: a randomized controlled trial. Alzheimer's Res. Ther. 11:3. doi: 10.1186/s13195-018-0454-z 30611286 PMC6320611

[B31] Kivipelto M. Mangialasche F. Ngandu T. (2018). Lifestyle interventions to prevent cognitive impairment, dementia and Alzheimer disease. Nat. Rev. Neurol. 14, 653–666. doi: 10.1038/s41582-018-0070-3 30291317

[B32] Knapp G. Hartung J. (2003). Improved tests for a random effects meta-regression with a single covariate. Stat. Med. 22, 2693–2710. doi: 10.1002/sim.1482 12939780

[B33] Law L. L. F. Barnett F. Yau M. K. Gray M. (2014). Effects of combined cognitive and exercise interventions on cognition in older adults with and without cognitive impairment: a systematic review. Ageing Res. Rev. 15, 61–75. doi: 10.1016/j.arr.2014.02.008 24632497

[B34] Li Z. Peng X. Xiang W. Han J. Li K. (2018). The effect of resistance training on cognitive function in the older adults: a systematic review of randomized clinical trials. Aging Clin. Exp. Res. 30, 1259–1273. doi: 10.1007/s40520-018-0998-6 30006762

[B35] Liao Y.-Y. Chen I.-H. Hsu W.-C. Tseng H.-Y. Wang R.-Y. (2021). Effect of exergaming versus combined exercise on cognitive function and brain activation in frail older adults: A randomised controlled trial. Ann. Phys. Rehabilit. Med. 64:101492. doi: 10.1016/j.rehab.2021.101492 33454398

[B36] Liao Y.-Y. Chen I.-H. Lin Y.-J. Chen Y. Hsu W.-C. (2019). Effects of virtual reality-based physical and cognitive training on executive function and dual-task gait performance in older adults with mild cognitive impairment: a randomized control trial. Front. Aging Neurosci. 11:162. doi: 10.3389/fnagi.2019.00162 31379553 PMC6646677

[B37] Liao Y.-Y. Tseng H.-Y. Lin Y.-J. Wang C.-J. Hsu W.-C. (2020). Using virtual reality-based training to improve cognitive function, instrumental activities of daily living and neural efficiency in older adults with mild cognitive impairment. Eur. J. Phys. Rehabilit. Med. 56, 47–57. doi: 10.23736/S1973-9087.19.05899-4 31615196

[B38] Lüdecke D. (2019). esc: Effect Size Computation for Meta Analysis (Version 0.5.1). Available online at: https://CRAN.R-project.org/package=esc

[B39] Lumsden J. Edwards E. A. Lawrence N. S. Coyle D. Munafó M. R. (2016). Gamification of cognitive assessment and cognitive training: a systematic review of applications and efficacy. JMIR Serious Games 4:e11. doi: 10.2196/games.5888 27421244 PMC4967181

[B40] Lyons E. J. Tate D. F. Ward D. S. Bowling J. M. Ribisl K. M. Kalyararaman S. (2011). Energy expenditure and enjoyment during video game play: differences by game type. Med. Sci. Sports Exerc. 43:1987. doi: 10.1249/MSS.0b013e318216ebf3 21364477 PMC3271952

[B41] Mancioppi G. Fiorini L. Rovini E. Zeghari R. Gros A. Manera V. . (2021). Innovative motor and cognitive dual-task approaches combining upper and lower limbs may improve dementia early detection. Sci. Rep. 11:7449. doi: 10.1038/s41598-021-86579-3 33811226 PMC8018979

[B42] Martel D. Lauzé M. Agnoux A. Fruteau De Laclos L. Daoust R. Émond M. . (2018). Comparing the effects of a home-based exercise program using a gerontechnology to a community-based group exercise program on functional capacities in older adults after a minor injury. Exper. Gerontol. 108, 41–47. doi: 10.1016/j.exger.2018.03.016 29577975

[B43] Mayer F. Scharhag-Rosenberger F. Carlsohn A. Cassel M. Müller S. Scharhag J. (2011). The intensity and effects of strength training in the elderly. Deutsches Ärzteblatt Int. 108:359. doi: 10.3238/arztebl.2011.0359 21691559 PMC3117172

[B44] Miyachi M. Yamamoto K. Ohkawara K. Tanaka S. . (2010). Mets in adults while playing active video games: a metabolic chamber study. Med. Sci. Sports Exerc. 42, 1149–1153. doi: 10.1249/MSS.0b013e3181c51c78 19997034

[B45] Miyake A. Friedman N. P. Emerson M. J. Witzki A. H. Howerter A. Wager T. D. (2000). The unity and diversity of executive functions and their contributions to complex “frontal lobe” tasks: a latent variable analysis. Cogn. Psychol. 41, 49–100. doi: 10.1006/cogp.1999.0734 10945922

[B46] Moret B. Nucci M. Campana G. (2022). Effects of exergames on mood and cognition in healthy older adults: a randomized pilot study. Front. Psychol. 13:1018601. doi: 10.3389/fpsyg.2022.1018601 36420381 PMC9676977

[B47] Mueller F. F. Byrne R. Andrés J. Patibanda R. (2018). “Experiencing the body as play,” in Proceedings of the 2018 CHI Conference on Human Factors in Computing Systems. doi: 10.1145/3173574.3173784

[B48] Mueller F. F. Edge D. Vetere F. Gibbs M. R. Agamanolis S. Bongers B. . (2011). “Designing sports: a framework for exertion games,” in Proceedings of the SIGCHI Conference on Human Factors in Computing Systems. doi: 10.1145/1978942.1979330

[B49] Ngandu T. Lehtisalo J. Solomon A. Levälahti E. Ahtiluoto S. Antikainen R. . (2015). A 2 year multidomain intervention of diet, exercise, cognitive training, and vascular risk monitoring versus control to prevent cognitive decline in at-risk elderly people (finger): a randomised controlled trial. Lancet 385, 2255–2263. doi: 10.1016/S0140-6736(15)60461-5 25771249

[B50] Nichols E. Steinmetz J. D. Vollset S. E. Fukutaki K. Chalek J. Abd-Allah F. . (2022). Estimation of the global prevalence of dementia in 2019 and forecasted prevalence in 2050: an analysis for the global burden of disease study 2019. Lancet Public Health 7, e105–e125. doi: 10.1016/S2468-2667(21)00249-8 34998485 PMC8810394

[B51] O'Donovan C. Hirsch E. Holohan E. McBride I. McManus R. Hussey J. (2012). Energy expended playing xbox kinect™ and wii™ games: a preliminary study comparing single and multiplayer modes. Physiotherapy 98, 224–229. doi: 10.1016/j.physio.2012.05.010 22898579

[B52] O'Dwyer S. T. Burton N. W. Pachana N. A. Brown W. J. (2007). Protocol for fit bodies, fine minds: a randomized controlled trial on the affect of exercise and cognitive training on cognitive functioning in older adults. BMC Geriatr. 7, 23–23. doi: 10.1186/1471-2318-7-23 17915035 PMC2094709

[B53] Owen A. M. Hampshire A. Grahn J. A. Stenton R. Dajani S. Burns A. . (2010). Putting brain training to the test. Nature 465, 775–778. doi: 10.1038/nature09042 20407435 PMC2884087

[B54] Peckham A. D. (2021). Why don't cognitive training programs transfer to real life? Three possible explanations and recommendations for future research. Behav. Ther. 44, 357–360. 35813267 PMC9262342

[B55] Peng Y. Wang Y. Zhang L. Zhang Y. Sha L. Dong J. . (2024). Virtual reality exergames for improving physical function, cognition and depression among older nursing home residents: a systematic review and meta-analysis. Geriatr. Nurs. 57, 31–44. doi: 10.1016/j.gerinurse.2024.02.032 38503146

[B56] Pustejovsky J. (2024). clubSandwich: Cluster-Robust (Sandwich) Variance Estimators with Small-Sample Corrections. R package version 0.5.11.

[B57] Qian Y. Schwartz A. Jung A. Zhang Y. Seitz U. Wilds G. . (2024). The influence of separate and combined exercise and foreign language acquisition on learning and cognition. Brain Sci. 14:572. doi: 10.3390/brainsci14060572 38928573 PMC11201889

[B58] Rieker J. A. Reales J. M. Muiños M. Ballesteros S. (2022). The effects of combined cognitive-physical interventions on cognitive functioning in healthy older adults: a systematic review and multilevel meta-analysis. Front. Hum. Neurosci. 16:838968. doi: 10.3389/fnhum.2022.838968 35399365 PMC8987130

[B59] Rogge A.-K. Röder B. Zech A. Nagel V. Hollander K. Braumann K.-M. . (2017). Balance training improves memory and spatial cognition in healthy adults. Sci. Rep. 7:5661. doi: 10.1038/s41598-017-06071-9 28720898 PMC5515881

[B60] Roig M. Nordbrandt S. Geertsen S. S. Nielsen J. B. (2013). The effects of cardiovascular exercise on human memory: a review with meta-analysis. Neurosci. Biobehav. Rev. 37, 1645–1666. doi: 10.1016/j.neubiorev.2013.06.012 23806438

[B61] Sanders L. M. Hortobágyi T. la Bastide-van Gemert S. van der Zee E. A. van Heuvelen M. J. G. (2019). Dose-response relationship between exercise and cognitive function in older adults with and without cognitive impairment: a systematic review and meta-analysis. PLoS ONE 14, 1–24. doi: 10.1371/journal.pone.0210036 30629631 PMC6328108

[B62] Schaefer S. Schumacher V. (2010). The interplay between cognitive and motor functioning in healthy older adults: findings from dual-task studies and suggestions for intervention. Gerontology 57, 239–246. doi: 10.1159/000322197 20980735

[B63] Sikkes S. A. Tang Y. Jutten R. J. Wesselman L. M. Turkstra F. Brodaty H. . (2021). Toward a theory-based specification of non-pharmacological treatments in aging and dementia: Focused reviews and methodological recommendations. Alzheimer's Dement. 17, 255–270. doi: 10.1002/alz.12188 33215876 PMC7970750

[B64] Smith G. E. Housen P. Yaffe K. Ruff R. Kennison R. F. Mahncke H. W. . (2009). A cognitive training program based on principles of brain plasticity: results from the improvement in memory with plasticity-based adaptive cognitive training (impact) study. J. Am. Geriatr. Soc. 57, 594–603. doi: 10.1111/j.1532-5415.2008.02167.x 19220558 PMC4169294

[B65] Srivastava S. Ahmad R. Khare S. K. Raza S. S. Prasad R. (2021). Alzheimer's disease and its treatment by different approaches: a review. Eur. J. Med. Chem. 216:113320. doi: 10.1016/j.ejmech.2021.113320 33652356

[B66] Stanmore E. Stubbs B. Vancampfort D. de Bruin E. D. Firth J. (2017). The effect of active video games on cognitive functioning in clinical and non-clinical populations: a meta-analysis of randomized controlled trials. Neurosci. Biobehav. Rev. 78, 34–43. doi: 10.1016/j.neubiorev.2017.04.011 28442405

[B67] Stanmore E. K. Mavroeidi A. De Jong L. D. Skelton D. A. Sutton C. J. Benedetto V. . (2019). The effectiveness and cost-effectiveness of strength and balance exergames to reduce falls risk for people aged 55 years and older in UK assisted living facilities: a multi-centre, cluster randomised controlled trial. BMC Med. 17:49. doi: 10.1186/s12916-019-1278-9 30813926 PMC6394073

[B68] Taylor L. M. Maddison R. Pfaeffli L. A. Rawstorn J. C. Gant N. Kerse N. M. (2012). Activity and energy expenditure in older people playing active video games. Arch. Phys. Med. Rehabil. 93, 2281–2286. doi: 10.1016/j.apmr.2012.03.034 22522217

[B69] Van Santen J. Dröes R.-M. Twisk J. W. Blanson Henkemans O. A. Van Straten A. Meiland F. J. (2020). Effects of exergaming on cognitive and social functioning of people with dementia: a randomized controlled trial. J. Am. Med. Direct. Assoc. 21, 1958–1967.e5. doi: 10.1016/j.jamda.2020.04.018 32651132

[B70] Viechtbauer W. (2005). Bias and efficiency of meta-analytic variance estimators in the random-effects model. J. Educ. Behav. Statist. 30, 261–293. doi: 10.3102/10769986030003261

[B71] Viechtbauer W. (2010). Conducting meta-analyses in R with the metafor package. J. Stat. Softw. 36, 1–48. doi: 10.18637/jss.v036.i03

[B72] Voinescu A. Papaioannou T. Petrini K. Fraser D. S. (2021). Exergaming for dementia and mild cognitive impairment. Cochr. Datab. System. Rev. 2024:CD013853. doi: 10.1002/14651858.CD013853 39319863 PMC11423707

[B73] Wimo A. Guerchet M. Ali G.-C. Wu Y.-T. Prina A. M. Winblad B. . (2017). The worldwide costs of dementia 2015 and comparisons with 2010. Alzheimer's Dement. 13, 1–7. doi: 10.1016/j.jalz.2016.07.150 27583652 PMC5232417

[B74] Wollesen B. Voelcker-Rehage C. (2014). Training effects on motor–cognitive dual-task performance in older adults. Eur. Rev. Aging Phys. Activ. 11, 5–24. doi: 10.1007/s11556-013-0122-z

[B75] World Health Organization (2023). Dementia. Available online at: https://www.who.int/news-room/fact-sheets/detail/dementia

[B76] Wu C. Yi Q. Zheng X. Cui S. Chen B. Lu L. . (2019). Effects of mind-body exercises on cognitive function in older adults: a meta-analysis. J. Am. Geriatr. Soc. 67, 749–758. doi: 10.1111/jgs.15714 30565212

[B77] Wu S. Ji H. Won J. Jo E.-A. Kim Y.-S. Park J.-J. (2023). The effects of exergaming on executive and physical functions in older adults with dementia: randomized controlled trial. J. Med. Internet Res. 25:e39993. doi: 10.2196/39993 36881445 PMC10031442

[B78] Yan J. Li X. Guo X. Lin Y. Wang S. Cao Y. . (2023). Effect of multicomponent exercise on cognition, physical function and activities of daily life in older adults with dementia or mild cognitive impairment: a systematic review and meta-analysis. Arch. Phys. Med. Rehabil. 104, 2092–2108. doi: 10.1016/j.apmr.2023.04.011 37142178

[B79] Yen H.-Y. Chiu H.-L. (2021). Virtual reality exergames for improving older adults' cognition and depression: a systematic review and meta-analysis of randomized control trials. J. Am. Med. Dir. Assoc. 22, 995–1002. doi: 10.1016/j.jamda.2021.03.009 33812843

[B80] Zhao Y. Feng H. Wu X. Du Y. Yang X. Hu M. . (2020). Effectiveness of exergaming in improving cognitive and physical function in people with mild cognitive impairment or dementia: systematic review. JMIR Serious Games 8:e16841. doi: 10.2196/16841 32602841 PMC7367532

