# Supplementary material for: Evaluating the cognitive impact of exergames on community-dwelling older adults beyond laboratory settings: a systematic review and meta-analysis
Source: Front Dement. 2026 Apr 10;5:1768487. doi: 10.3389/frdem.2026.1768487 (PMC13105986; doi:10.3389/frdem.2026.1768487)
Supplement: Supplementary file 1 [file Data_Sheet_1.pdf]

Supplementary Material

1 SUPPLEMENTARY MATERIAL DATA

1.1 Search Terms

PubMed: ("exercise game" OR "exergame" OR "active video game" OR "virtual reality" OR "augmented reality") AND ("attention" OR "memory" OR "executive function" OR "processing speed" OR "cognition" OR "cognitive") AND ("older adults" OR "seniors" OR "elderly")

ScienceDirect: ("exercise game" OR "exergame" OR "active video game" OR "virtual reality" OR "augmented reality") AND ("attention" OR "memory" OR "executive function" OR "processing speed" OR "cognitive" OR "cognition") AND ("older adults" OR "seniors" OR "elderly")

SpringerLink: ("exergame" OR "active video game" OR "virtual reality" OR "augmented reality") AND ("attention" OR "memory" OR "executive function" OR "processing speed") AND ("older adults" OR "seniors" OR "elderly")

ACM: [[All: "exergame"] OR [All: "exercise game"] OR [All: "active video game"] OR [All: "virtual reality"] OR [All: "augmented reality"]] AND [[All: and] OR [All: "older adults"] OR [All: "seniors"] OR [All: "elderly"]] AND [[All: and "attention"] OR [All: "memory"] OR [All: "executive function"] OR [All: "processing speed"] OR [All: "cognition"] OR [All: "cognitive"]]

IEEE: ("exercise game" OR "exergame" OR "active video game" OR "virtual reality" OR "augmented reality") AND ("attention" OR "memory" OR "executive function" OR "processing speed" OR "cognition" OR "cognitive") AND ("older adults" OR "seniors" OR "elderly")

1.2 Figures and Tables

Author	Outcomes	Cognition	Effect Size ID
Gschwind et al. (2015a)	stroop_sec	processing speed	1
Gschwind et al. (2015a)	stroop_error	executive	2
Gschwind et al. (2015a)	stroop_efficiency	executive	3
Gschwind et al. (2015a)	digitSpan_backwards	memory	4
Gschwind et al. (2015b)	TMT-A	attention	5
Gschwind et al. (2015b)	TMT-B	executive	6
Gschwind et al. (2015b)	digitalSpan_coding_correct	memory	7
Gschwind et al. (2015b)	digitalSpan_backwards	memory	8
Gschwind et al. (2015b)	ANT-reactionTime(ms)	processing speed	9
Gschwind et al. (2015b)	ANT-alert(ms)	attention	10
Gschwind et al. (2015b)	ANT-orient(ms)	attention	11
Gschwind et al. (2015b)	ANT-conflict(ms)	attention	12
Gschwind et al. (2015b)	stroop_intrusions(n)	executive	13
Gschwind et al. (2015b)	stroop_efficacy(colourWords)	executive	14
Martel et al (2018)	MoCA	global	15
Karssemeijer et al (2019)	TMT-B	executive	16
Karssemeijer et al (2019)	stroop_speedaccuracy	executive	17
Karssemeijer et al (2019)	stroop_colorWord_sec	processing speed	18
Karssemeijer et al (2019)	stroop_colorWord_err	executive	19
Karssemeijer et al (2019)	letterFluency	verbal	20
Karssemeijer et al (2019)	RuleShift	executive	21
Karssemeijer et al (2019)	TMT-A	attention	22
Karssemeijer et al (2019)	stroop_wordReading_sec	processing speed	23
Karssemeijer et al (2019)	stroop_colorName_sec	processing speed	24
Karssemeijer et al (2019)	locationLearn_trail	memory	25
Karssemeijer et al (2019)	locationLearn_delay	memory	26
Karssemeijer et al (2019)	digitSpan	memory	27
Karssemeijer et al (2019)	spatialSpan	memory	28
Stanmore et al (2019)	ACE III	executive	29
Santen MSc et al (2020)	MMSE	executive	30
Santen MSc et al (2020)	TMT-A	attention	31
Santen MSc et al (2020)	TMT-B	executive	32
Liao et al. (2020)	MoCA	global	33
Liao et al. (2020)	EXIT-25	global	34
Liao et al. (2020)	CVVLT_imRecall	memory	35
Liao et al. (2020)	CVVLT_delayRecall	memory	36
Gouvela et al (2020)	COGTEL_WM	executive	37
Gouvela et al (2020)	COGTEL_STM	memory	38
Gouvela et al (2020)	COGTEL_VF	verbal	39
Gouvela et al (2020)	COGTEL_IR	executive	40
Gouvela et al (2020)	COGTEL_LTM	memory	41
Gouvela et al (2020)	COGTEL_total	global	42
Liao et al. (2021)	MoCA	global	43
Liao et al. (2021)	EXIT-25	global	44
Liao et al. (2021)	CCVLT_verbalWM	memory	45
Liao et al. (2021)	CCVLT_delayRecall	memory	46
Liao et al. (2021)	stroop_colourWord(n)	executive	47
Liao et al. (2021)	TMT-B	executive	48
Liao et al. (2021)	N-back_1	executive	49
Liao et al. (2021)	N-back_2	executive	50
Wu et al (2023)	Flanker_congRT	executive	51
Wu et al (2023)	Flanker_incongRT	executive	52

Table S1. The specific cognitive domain of the measurements used in each study marked with the unique effect size id. TMT-A/TMT-B: Trial Making Test, ANT:Attention Network Test, MoCA: Montreal Cognitive Assessment, MMSE: Mini-Mental State Examination, ACE III: Addenbrooke's Cognitive Examination, CVVLT: The Chinese version of the Verbal Learning Test, COGTEL: Cognitive Telephone Screening Instrument, Exit-25: Executive Interview, Three effect sizes were identified as both statistical outliers and highly influential cases: (1) Stroop test reaction time from Gschwind et al. (2015a), (2) Attention Network Test (ANT) reaction time from Gschwind et al. (2015b), and (3) COGTEL working memory from Gouvela et al (2020). Detection methods included Baujat plots to identify studies contributing disproportionately to overall heterogeneity, funnel plot visual inspection for extreme values, and standardized residual analysis. Influence analysis using the leave-one-out method confirmed that removal of these three effect sizes substantially reduced between-study heterogeneity while maintaining a similar pooled effect estimate.

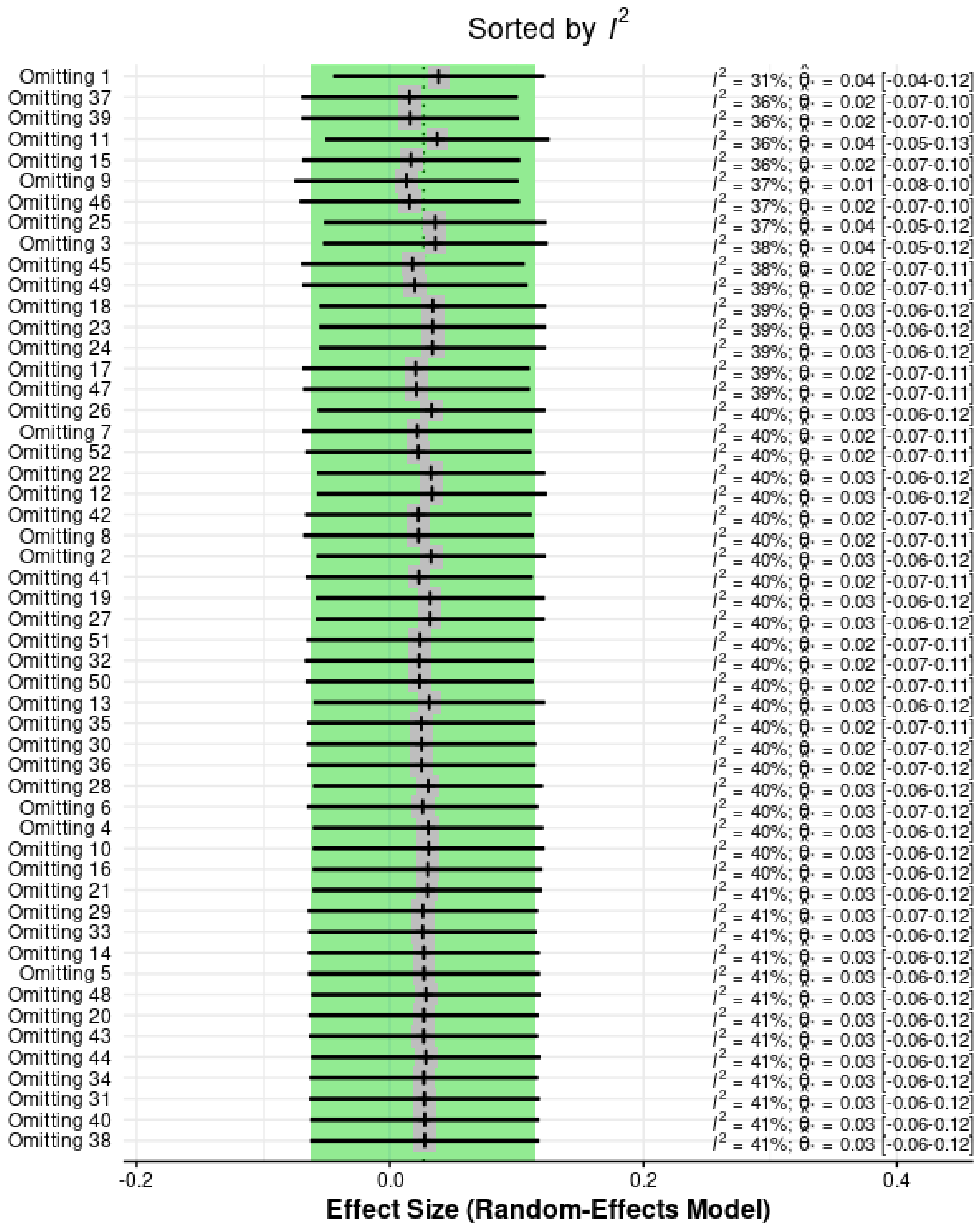

Figure S1. The forest plot visualized the leave-and-out method, which shows the lowest I² value when omitting the effect size id one at a time.

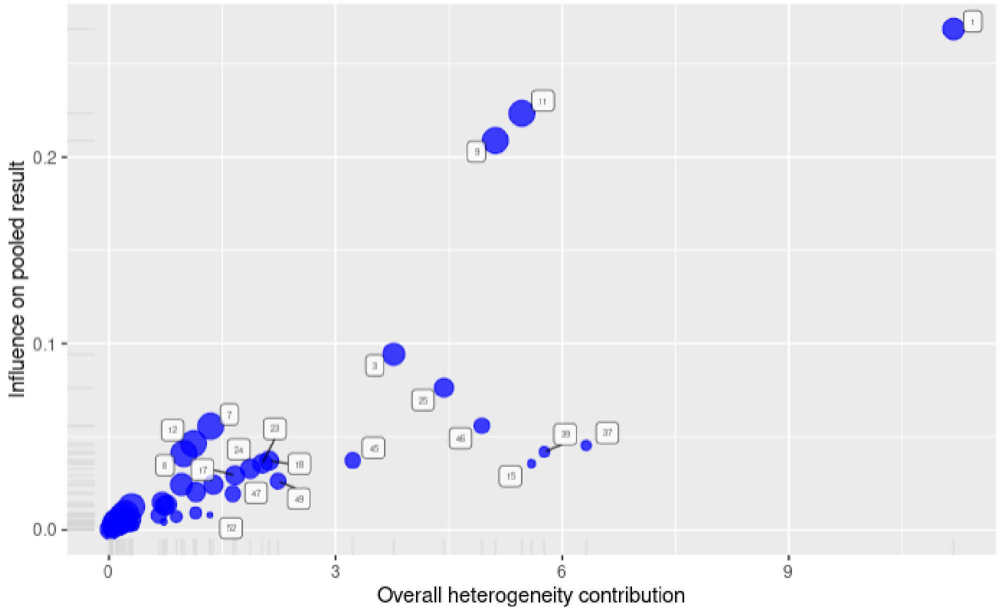

Figure S2. This Baujat plot visualized the influential cases based on the effect size and marked with effect size IDs.

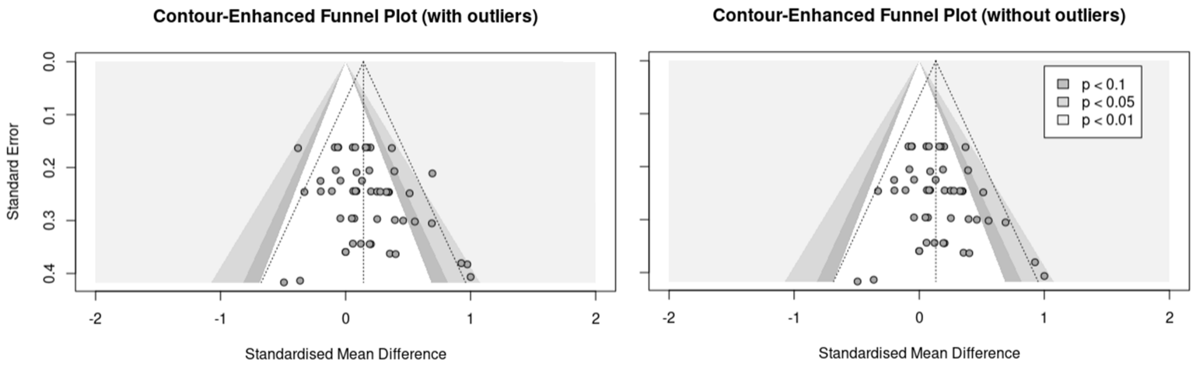

Figure S3. Funnel plot shows the symmetrical pattern of the effect sizes. The X-axis indicates the standardized mean difference, and the Y-axis indicates the standard error of the effect size.
