# Supplementary material for: Evaluating the cognitive impact of exergames on community-dwelling older adults beyond laboratory settings: a systematic review and meta-analysis
Source: Front Dement. 2026 Apr 10;5:1768487. doi: 10.3389/frdem.2026.1768487 (PMC13105986; doi:10.3389/frdem.2026.1768487)
Supplement: Supplementary file 2 [file Data_Sheet_2.pdf]

Supplementary Material

1 SUPPLEMENTARY TABLES

1.1 Table S2. Study 1-5 Exergame Interventions: Technical Features, Delivery, Attrition, and Safety

1.2 Table S3. Study 6-10 Exergame Interventions: Technical Features, Delivery, Attrition, and Safety

Table S1. Exergame Interventions: Technical Features, Delivery, Attrition, and Safety

Author	Exergame Type & Key Features	Technical Setup	Supervision & Delivery	Attrition (%)	Safety/Adverse Events
Gschwind et al (2015a)	**Step Mat Training:** 4 exergames combined pmulti-directional stepping with cognitive challenges<br>●Modified StepMania: Timed and coordinated stepping according to arrows on screen that differed in direction and drift speed<br>●Stepper: Rapid stepping in four directions<br>●Trail-stepping: Stepping to connect numbers and letters<br>●Tetris-stepping: Stepping to rotate and control blocks	Pressure-sensitive electronic mat with 6 arrows (front, side, back)	Unsupervised home-based	17% (8/47). Death (n=1), health (n=5), personal (n=2)	No adverse events
Gschwind et al (2015b)	**iStoppFalls Kinect:** 3 balance games (Bumble Bee Park, Hills & Skills, Balance Bistro) + 5 Otago strength exercises<br>●Walking, weight-shifting, knee bending, multi-directional stepping<br>●Avatar on TV controlled by movements,<br>●Navigation through system using gestures, voice control, or tablet	PC, Google TV box, Kinect sensor, Philips mobility monitor, Nexus tablet. Home installation	Unsupervised home with remote monitoring, monthly calls	19.2% (15/78). Health (n=5), technical (n=4), going away (n=2), other (n=4)	No adverse events. 24 total falls (8 intervention, 15 control), none from intervention
Martel et al (2018)	**Jintronix:** Multicomponent<br>●Cardio (20min): Butt kicks, high knees, lateral launches<br>●Strength/balance (20min): Squats, leg extension, core<br>●Real-time visual/audio feedback<br>●Personalized: Speed, ROM, precision, reps, duration	Kinect on participant's TV. Web portal for remote monitoring	1:1 home. Kinesiologist at 6 visits + phone calls. Remote monitoring	11.1% (2/18). Poor health (n=1), no interest (n=1)	No adverse events. Safe for all
Karssemeijer et al (2019)	**Bike Labyrinth:**<br>●Bike + video screen with digital routes<br>●Cycling pace controls video speed<br>●7 cognitive levels: Response inhibition, task switching, processing speed<br>●Progressive difficulty maintains challenge	Stationary bike connected to video screen	1:1 by trained students/research assistants at community centers	10.5% (4/38). Health (n=3), fatigue (n=1)	No serious adverse events
Stanmore et al (2019)	**OTAGO/FaME Exergames:** Co-designed<br>●Lower/upper limb exercises matched to games<br>●Changeable games for interest<br>●Tailored to ability, preference<br>●Visual/audio feedback<br>●Gamification: Levels, points, progress	Kinect v2, laptop, TV. Sensor 1m height, participant 2-3m away. Space: $>10m^2$	1:1 by physiotherapist/trained assistant in facility communal rooms. 30min training	12.5% (7/56). Medical (n=5), family (n=1), loss of interest (n=1)	No adverse events

Table S2. Exergame Interventions: Technical Features, Delivery, Attrition, and Safety

Author	Exergame Type & Key Features	Technical Setup	Supervision & Delivery	Attrition (%)	Safety/Adverse Events
Santen et al (2020)	**Interactive Cycling:** Commercial systems (DiFiets, Fietslabyrint, PraxFit, SilverFit Mile)<br>●Bike + screen with routes<br>●Participant selects route<br>●Cycling pace controls speed<br>●Mimics outdoor cycling<br>●Social: Discussion of images	Stationary bike + screen.	Day care staff. Staff: 1hr training. Free provider training not used	28.7% (21/73). Care home (n=8), wants stop (n=7), caregiver stop (n=3), no dementia (n=2), death (n=1), health (n=1)	No adverse events
Gouveia et al (2020)	**5 Custom Games** (Madeira culture):<br>●Grape Stomping: Stepping for wine-making (aerobic)<br>●Rabelos: Rowing virtual boat (upper strength)<br>●Exermusic: Musical movement (lower strength/flexibility)<br>●Toboggan: Postural stability/trunk (balance)<br>●Exerpong: Paddle control (aerobic/motor)<br>●Points, bonuses, progressive challenges	Floor projection 2.5m×3m PVC. KinectV2 tracking 25 joints. At senior gymnasium	1:1 by 2 sport science professionals. 1-month training. Week 0 familiarization	21.1% (4/19). Lost interest pre-intervention (n=1), health (n=3). Only involuntary reasons	No adverse events or side effects
Liao et al (2020)	**VR Physical-Cognitive:** Kinect + HTC VIVE<br>●Physical: Tai chi, resistance, aerobic stepping<br>●Functional: Window cleaning, goldfish scooping, obstacles, stairs<br>●Cognitive: Navigate MRT, find stores, checkout clerk<br>●Avatar imitation, real-time feedback	Kinect full-body 3D mapping. HTC VIVE headset + controllers in 4m×3m space	Groups (3-4) by 2 physical therapists	14.3% (3/21). Health (n=1), low motivation (n=2)	No adverse events
Liao et al (2021)	**Kinect Multi-Domain:** 8 activities<br>●Swimming: 3D pool, full-body, cardio<br>●Running/Stepping: Athletic field, tracking<br>●Obstacles: Crossing, stairs, balance<br>●Tai Chi: Circular motions, breathing, virtual guide<br>●Window/Firework/Goldfish/Leg games<br>●Infrared tracking, real-time feedback	Kinect infrared motion capture. Full-body 3D virtual mapping	Groups (3-4) by 2 physical therapists	19.4% (6/31). Low motivation (n=2), scheduling (n=2), no fNIRS data (n=2)	No adverse effects
Wu et al (2023)	**ExerHeart Mat Game:**<br>●Running/jumping on mat controls avatar<br>●Avoid obstacles, collect items<br>●Speed adjusted by movement intensity	Pressure-sensitive mat 950×1300×1700mm. Screen with game/avatar	Supervised (level not specified)	50% (13/26). Moved daycare (n=8, 62%), hospitalized (n=2, 15%), refused (n=3, 23%)	Not mentioned
